# Evolution of (*S*)-2-Aminobutanol Synthesis from Chemical Resolution to Biomanufacturing

**DOI:** 10.3390/ijms27209203

**Published:** 2026-10-16

**Authors:** Bing-Bing Wang, Chang-Dong Chen, Yong-Jun Han, Wei-Shuang Tong, Hua-Shan Gao, Wei Ma, Jun-Yang Liu, Jian-Zhong Xu

**Affiliations:** 1 School of Materials Science and Engineering, Pingdingshan University, Pingdingshan 467000, China; 2 School of Environmental and Chemical Engineering, Xi’an Polytechnic University, Xi’an 710048, China; 3 The Key Laboratory of Carbohydrate Chemistry and Biotechnology, Ministry of Education, School of Biotechnology, Jiangnan University, Wuxi 214122, China

**Keywords:** (*S*)-2-aminobutanol, chiral resolution, asymmetric catalysis, transaminase, whole-cell factory, synthetic biology

## Abstract

As a pivotal chiral precursor for the synthesis of the antituberculosis drug ethambutol and antiepileptic drugs such as levetiracetam, Chiral (*S*)-2-aminobutanol requires high optical purity for clinical safety. This review systematically summarizes the evolution and recent advances in its synthetic routes. Traditional chemical methods, including the 1-nitropropane and 1-butene epoxidation routes, suffer from severe safety hazards and heavily rely on downstream chiral resolution. However, both chemical crystallization and enzymatic kinetic resolution are constrained by a 50% theoretical yield ceiling and incur heavy environmental burdens. To break this bottleneck, in vitro multienzyme cascades utilizing amine dehydrogenases (AmDHs) and ω-transaminases (ω-TAs) have emerged, achieving 100% theoretical yield from prochiral precursors and >99% enantiomeric excess (ee) driven by a distinct dual-pocket active site model. Furthermore, synthetic biology has enabled de novo biosynthesis via whole-cell cell factories. It should be noted that the recently reported 45.3 g·L^−1^ titer refers to the upstream amino acid precursor L-2-aminobutyric acid (L-2-ABA), not to the final (*S*)-2-aminobutanol product. To overcome bottlenecks like the intracellular toxicity of the intermediate 2-ketobutyrate (2-OBA), strategies including efflux pump engineering (e.g., *rhtA*) and quorum sensing-based dynamic regulation have successfully boosted the fermentation titer to 45.3 g·L^−1^. Finally, the industrialization potential of these generations of technologies is evaluated, highlighting that the transition toward in vitro continuous-flow microreactors and a hybrid whole-cell + cell-free *architecture*, combined with the integration of AI into whole-cell factories, represents a future trend for green and low-carbon manufacturing.

## 1. Introduction

Chiral (*S*)-2-aminobutanol is a valuable amino alcohol widely used in the fine chemical and pharmaceutical industries and serves as an important chiral building block for the synthesis of various bioactive molecules and active pharmaceutical ingredients (APIs). In particular, it is a key chiral intermediate for the synthesis of ethambutol, a first-line antituberculosis drug [1]. The pharmacological activity and toxicity of ethambutol are strongly dependent on its stereochemical configuration. Whereas the therapeutically active stereoisomer exhibits potent antituberculosis activity, its enantiomer has been associated with severe adverse effects, including visual impairment and blindness. Therefore, stringent stereochemical control of the chiral precursor is essential for the synthesis of ethambutol [2]. In addition, related chiral C4 amino compounds and their derivatives are important intermediates in the manufacture of antiepileptic drugs such as levetiracetam and brivaracetam [3]. More broadly, chiral amino alcohols and related chiral amine building blocks are widely found in the molecular scaffolds or synthetic routes of pharmaceuticals, including anti-HIV agents such as elvitegravir [4], and also have important applications in agrochemicals, polymers, dyes, emulsifiers, plasticizers, and other high-value fine chemicals [5].

The growing demand for cost-effective chiral building blocks with high optical purity has stimulated considerable interest in more efficient manufacturing technologies. Although pharmaceuticals such as ethambutol and levetiracetam are now widely available as generic drugs, their production costs can remain relatively high because of limitations associated with conventional synthetic processes, particularly the additional costs of chiral resolution. In many developing regions, such manufacturing costs may contribute to limited drug accessibility and impose a substantial economic burden on patients. Consequently, the development of low-cost, efficient, and environmentally sustainable routes for the production of optically pure chiral intermediates has become an important objective in pharmaceutical manufacturing and global healthcare [6].

Conventionally, the synthesis of chiral amino alcohols often involves harsh reaction conditions, expensive transition-metal catalysts, toxic reagents, organic solvents, and multiple protection and deprotection steps. Moreover, insufficient regio- and enantioselectivity remains a major limitation of many chemical routes. When racemic products are generated, subsequent kinetic or optical resolution is required, for which the maximum theoretical yield is intrinsically limited to 50% [7]. These limitations have driven increasing interest in green biomanufacturing processes capable of operating at high substrate concentrations while maintaining high enantiomeric purity. Compared with conventional chemical synthesis, biocatalysis offers several advantages, including mild reaction conditions, high selectivity, improved atom efficiency, and reduced generation of environmentally hazardous waste. Among the available biocatalysts, ω-TAs and amine dehydrogenases (AmDHs) have emerged as powerful tools for the asymmetric synthesis of chiral amines and amino alcohols. ω-TAs catalyze asymmetric transamination using an appropriate amine donor, although unfavorable reaction equilibria may necessitate the use of excess amine donor or efficient co-product removal. In contrast, AmDHs can directly catalyze the asymmetric reductive amination of prochiral ketones using inexpensive ammonia as the amino donor and NAD(P)H as the reducing equivalent [8]. Engineered AmDHs have enabled the synthesis of (*S*)-configured chiral amino alcohols with conversions of up to 99% and enantiomeric excess (ee) values exceeding 99% [9]. By avoiding racemate formation and minimizing the generation of hazardous waste, such biocatalytic strategies provide a promising green and sustainable platform for the manufacture of optically pure pharmaceutical intermediates and other high-value fine chemicals.

Unlike previous reviews focusing broadly on chiral amines and amino alcohols, this review specifically examines the evolution of (*S*)-2-aminobutanol production, from conventional chemical synthesis and chiral resolution to enzymatic catalysis and whole-cell biomanufacturing. Particular emphasis is placed on the technological transitions between synthetic strategies, the integration of enzyme stereoselectivity with metabolic pathway engineering, and the comparative assessment of industrial feasibility based on yield, enantiomeric excess, productivity, and downstream processing requirements. By combining historical development, mechanistic insights, and process-level evaluation, this review provides a comprehensive perspective on current challenges and future opportunities for sustainable (*S*)-2-aminobutanol production.

## 2. Conventional Chemical Synthesis Routes and Process Limitations

**Figure 1:**
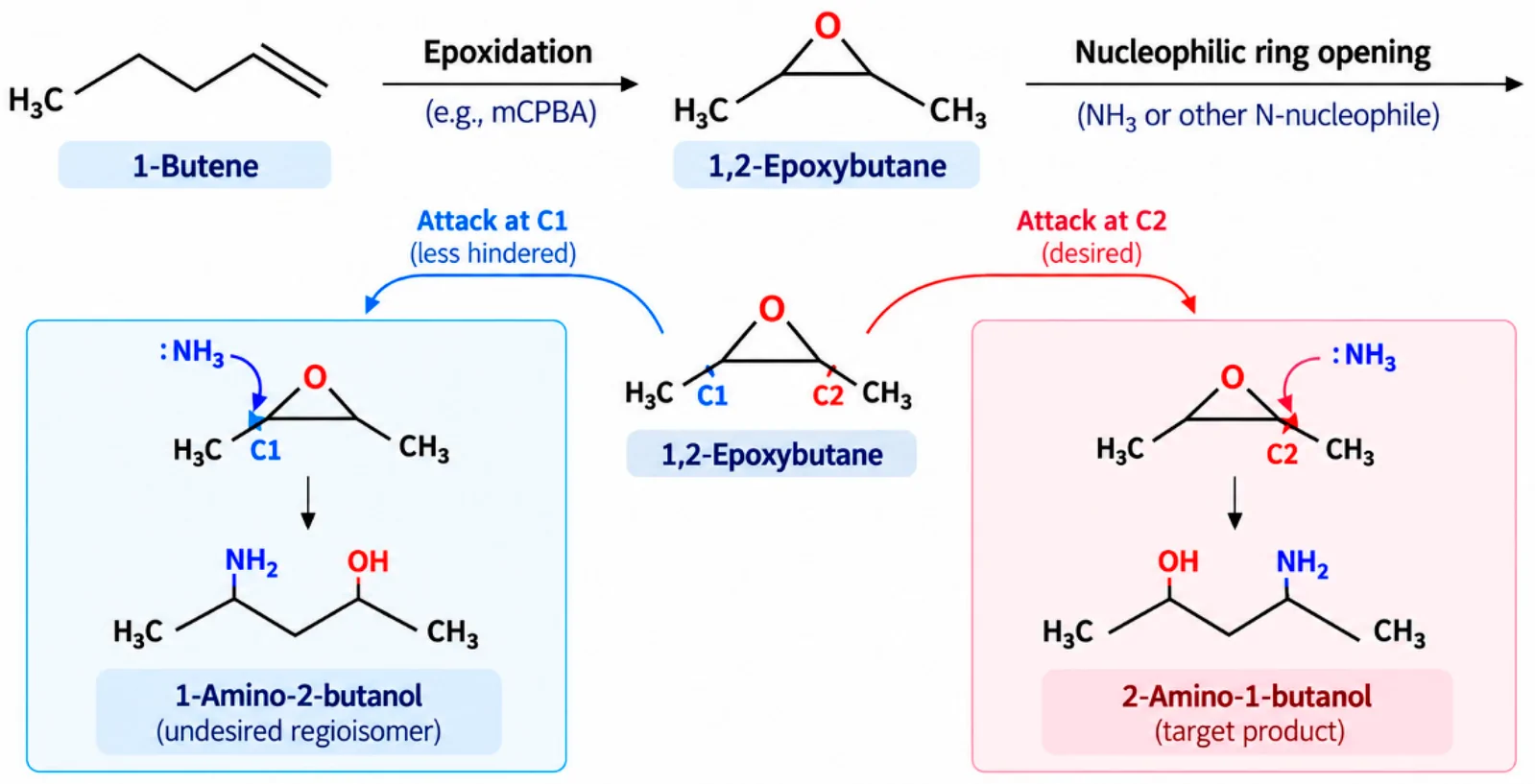
Schematic representation of the 1-butene-1,2-epoxybutane route and regioselective ring opening for 2-amino-1-butanol synthesis.

Chemical synthesis has historically played an important role in the production of chiral amines and amino alcohols. Nevertheless, achieving both high yield and high stereoselectivity by purely chemical approaches is often challenging. Conventional routes to chiral amines frequently rely on transition-metal-catalyzed hydrogenation or stoichiometric hydride reducing agents, and some systems require elevated hydrogen pressures, relatively high catalyst or additive loadings, and additional substrate-activation steps [10]. Moreover, many asymmetric synthetic strategies employ N-acyl, N-phosphinoyl, N-aryl, or N-benzyl groups as protecting or activating groups, followed by an additional deprotection step, thereby reducing overall step economy [10]. Chemical synthesis of amino alcohols may similarly involve strong reducing agents or organometallic reagents, such as DIBAL-H and LiAlH_4_, together with protecting-group manipulations and potential problems associated with stereochemical control and racemization [11]. For a structurally simple product such as 2-amino-1-butanol, for which high optical purity is required, reducing the number of reaction steps and minimizing catalyst and reagent consumption while establishing stereoselectivity at the point of synthesis therefore remain important objectives for process development (Figure 1).

### 2.1. 1-Nitropropane Route via Henry Reaction and Catalytic Hydrogenation

A representative chemical route to 2-amino-1-butanol starts from 1-nitropropane and formaldehyde. The first step involves a Henry reaction. Under basic conditions, deprotonation at the α-carbon of 1-nitropropane generates a nucleophilic nitronate anion, which subsequently undergoes nucleophilic addition to the carbonyl carbon of formaldehyde. Formation of the new C-C bond yields 2-nitro-1-butanol as the key intermediate. In the second step, the nitro group is reduced by catalytic hydrogenation to afford 2-amino-1-butanol.

From a process perspective, a major burden of this route lies in the hydrogenation step. Conventional chemical routes to amines commonly employ heterogeneous catalysts such as Raney Ni, Pd/C, or Pt/C under pressurized hydrogen, with the optimal catalyst, hydrogen pressure, and reaction time being highly substrate dependent [10]. Scale-up therefore requires careful management of gas–liquid–solid mass transfer, hydrogen handling, catalyst separation and recovery, and competing side reactions. More importantly, this synthetic sequence does not intrinsically establish the desired stereochemistry. Consequently, production of optically pure (*S*)-2-amino-1-butanol requires either an additional asymmetric induction strategy or subsequent resolution.

It should be noted that references [10,11] provide general evidence regarding conventional chemical routes to chiral amines and amino alcohols but do not report specific industrial hydrogenation conditions or thermal-safety parameters for 2-nitro-1-butanol. Therefore, quantitative claims regarding catalyst loading, hydrogen pressure, or thermal decomposition of this particular intermediate should be supported by dedicated process or safety studies.

### 2.2. 1-Butene to 1,2-Epoxybutane Route via Epoxidation and Regioselective Ring Opening

Another classical strategy for constructing vicinal amino alcohols involves epoxidation of an alkene followed by nucleophilic ring opening with a nitrogen-containing nucleophile. Starting from 1-butene, epoxidation affords 1,2-epoxybutane, which can subsequently undergo ring opening with ammonia or another nitrogen nucleophile to generate the amino alcohol framework.

The principal challenge of this route lies not in epoxide formation itself but in controlling the regioselectivity of nucleophilic ring opening of the unsymmetrical terminal epoxide. For 1,2-epoxybutane, nucleophilic attack at the less hindered terminal carbon (C1) leads to 1-amino-2-butanol, whereas attack at C2 affords the desired 2-amino-1-butanol. Efficiently directing nucleophilic attack toward C2 is therefore critical for selective formation of the target regioisomer.

Harris et al. [12,13] systematically investigated nucleophilic ring opening of terminal and internal epoxides. With simple lithium amides as nucleophiles, terminal epoxides showed a strong preference for attack at the terminal carbon and, under some conditions, afforded a single regioisomer. In contrast, Lewis-acid-catalyzed reactions of styrene oxide produced both regioisomeric amino alcohols, with regioisomer ratios ranging from approximately 60:40 to 85:15 and favoring the product derived from terminal attack [13]. These observations demonstrate that the regioselectivity of epoxide ring opening is highly dependent on the substrate, nucleophile, and catalytic environment. Importantly, the study by Harris et al. did not directly examine the reaction of 1,2-epoxybutane with ammonia; therefore, the reported regioisomer ratios should not be interpreted as quantitative product distributions for that specific system.

Classical epoxide–amine ring-opening reactions may also suffer from variable yields, long reaction times, relatively forcing conditions, and the requirement for excess amine [13]. In the context of 2-amino-1-butanol production, formation of positional isomers such as 1-amino-2-butanol would substantially increase the downstream purification burden. Because these regioisomers possess closely related structures and physicochemical properties, controlling regioselectivity during the reaction is generally more attractive than relying on extensive downstream separation.

### 2.3. Economic and Environmental Limitations of Resolution with the 50 Percent Theoretical Yield Ceiling

When chemical synthesis lacks effective stereochemical control, racemic products are obtained and an additional resolution step is required to isolate (*S*)-2-amino-1-butanol. Conventional approaches include diastereomeric salt crystallization, chiral chromatography, and enzymatic kinetic resolution. Nugent and El-Shazly noted that classical resolution and preparative chiral HPLC remain alternatives for chiral amines that cannot be efficiently accessed by direct asymmetric synthesis, although these approaches are unfavorable from a green-chemistry perspective [10].

Classical salt resolution is based on the formation of diastereomeric salts between a racemic amine and a chiral acid, followed by selective crystallization arising from differences in solubility. Achieving high optical purity can require multiple unit operations, including crystallization, filtration, liberation of the free amine, extraction, and solvent recovery. Consequently, resolution increases solvent, acid/base, and energy consumption and generates additional salt-containing and organic waste streams. More fundamentally, the maximum theoretical yield of the desired enantiomer in an ideal kinetic resolution is limited to 50% in the absence of in situ conversion of the undesired enantiomer [14]. It should be noted that this 50% ceiling refers to theoretical yield (maximum fraction of racemic substrate that can be converted into the desired enantiomer), not to atom economy in the strict Trost sense (the proportion of reactant atoms incorporated into the desired product), although the two metrics can be related in specific process contexts.

Across the conventional chemical routes surveyed in this section—whether the Henry reaction (Section 2.1), the epoxide route (Section 2.2), or alternative strategies such as the titanium-mediated cross-coupling of imines with aldehydes or ketones [15]—the central limitation is therefore not simply insufficient reaction yield; rather, insufficient selectivity at the synthetic stage transfers the burden to downstream separation and resolution.

The central limitation of conventional chemical routes is therefore not simply insufficient reaction yield; rather, insufficient selectivity at the synthetic stage transfers the burden to downstream separation and resolution. This limitation has provided a major impetus for the development of asymmetric biocatalysis. Highly stereoselective enzymes, including transaminases and amine dehydrogenases, can establish the desired stereocenter directly, thereby minimizing the formation of undesired enantiomers and regioisomers, improving substrate utilization, and simplifying downstream processing.

## 3. Chiral Resolution of Racemic 2-Amino-1-Butanol

Because conventional chemical synthesis generally affords racemic 2-amino-1-butanol, early industrial processes relied on subsequent chiral resolution to obtain (*S*)-2-amino-1-butanol with high optical purity. Among the approaches developed for this purpose, crystallization-based chemical resolution and enzymatic kinetic resolution represent two of the most established strategies. Nevertheless, when evaluated from the perspectives of modern green chemistry, process efficiency, and atom economy, both approaches exhibit inherent limitations (Table 1).

### 3.1. Crystallization-Based Chemical Resolution

Crystallization-based resolution is one of the classical approaches for obtaining optically enriched amino alcohols and commonly employs inexpensive, naturally derived L-tartaric acid as a chiral resolving agent. The physicochemical basis of this method is the conversion of a pair of enantiomers with essentially identical bulk physicochemical properties into diastereomeric salts with different solubilities and crystallization behaviors. In a suitable methanol–water or ethanol–water solvent system, racemic 2-amino-1-butanol undergoes acid–base salt formation with enantiopure L-tartaric acid, generating a pair of diastereomeric salts that differ in crystal packing and intermolecular hydrogen-bonding interactions.

In industrial crystallization, a half-equivalent resolution strategy based on the Pope–Peachey principle may be employed to reduce the consumption of the resolving agent. Careful control of supersaturation, nucleation, crystal growth, cooling profile, and solvent composition is essential for achieving both satisfactory recovery and high optical purity. In particular, the water content of the crystallization medium can influence the hydrogen-bonding network and relative solubilities of the diastereomeric salts. Consequently, inappropriate solvent composition may reduce either the crystallization yield or the enantiomeric purity of the isolated product.

Despite its technical maturity, L-tartaric-acid-based resolution has intrinsic limitations in terms of green chemistry and atom economy. As discussed by Huisman et al. [16] and related studies, conventional resolution is fundamentally a separation process rather than an asymmetric synthesis. In the absence of efficient in situ racemization, the theoretical maximum recovery of the desired (*S*)-enantiomer is limited to 50%, leaving the remaining (*R*)-enantiomer unutilized unless an additional recycling strategy is introduced.

To improve material utilization, racemization of the undesired enantiomer using transition-metal catalysts, including Ru- or Ir-based systems, or strongly basic conditions at elevated temperatures has been explored [17]. However, such racemization processes may require demanding reaction conditions and can promote dehydration, deamination, or other degradation pathways of amino alcohol substrates, thereby increasing both energy consumption and process complexity.

A further drawback of classical salt resolution is the burden associated with downstream processing. After isolation of the desired diastereomeric salt, a strong base such as NaOH is generally required to liberate free (*S*)-2-amino-1-butanol, followed by extraction and solvent recovery. Repeated crystallization, filtration, neutralization, extraction, and solvent recycling therefore increase the consumption of acids, bases, solvents, and energy while generating additional salt-containing and organic waste streams. These features substantially reduce the overall process efficiency and increase the environmental burden of conventional resolution.

### 3.2. Enzymatic Kinetic Resolution

To reduce the solvent consumption, energy demand, and operational complexity associated with chemical resolution, enzymatic kinetic resolution (KR) has been investigated for racemic 2-amino-1-butanol and related amino alcohols. Lipases and acylases are among the most extensively studied biocatalysts for this purpose because their asymmetric active sites can discriminate between enantiomeric substrates and selectively transform one enantiomer while leaving the other largely unreacted.

Lipase-catalyzed kinetic resolution generally relies on selective N- or O-acylation in a non-aqueous medium using a suitable acyl donor. Francalanci et al. [18] demonstrated that lipases can catalyze highly enantioselective acylation of racemic amino alcohols in organic media. In such systems, one enantiomer is preferentially converted into an amide or ester derivative, whereas the other remains predominantly unreacted. The resulting difference in polarity and chemical functionality facilitates downstream separation, and high enantiomeric enrichment can be achieved under appropriately optimized conditions.

Acylases have also been investigated for the resolution of amino-alcohol derivatives. Fadnavis et al. [21] reported the use of immobilized penicillin G acylase (PGA) for the optical resolution of an N-phenylacetyl derivative of racemic 2-amino-1-butanol, demonstrating high enantioselectivity. Subsequent process development further explored immobilized PGA in continuous kinetic-resolution systems. The use of immobilized enzymes in packed-bed reactors can facilitate catalyst reuse, continuous operation, and improved volumetric productivity, thereby offering potential advantages over conventional batch resolution [22].

### 3.3. From Kinetic Resolution to Dynamic Kinetic Resolution

Despite the high stereoselectivity attainable with lipases and acylases, conventional enzymatic KR remains intrinsically constrained by a maximum theoretical yield of 50% for the desired enantiomer [19]. Overcoming this limitation requires dynamic kinetic resolution (DKR), in which the selective enzymatic transformation is coupled with simultaneous in situ racemization of the unreacted enantiomer. In principle, continuous interconversion of the two enantiomers allows the entire racemic substrate pool to be converted into a single stereoisomer, increasing the theoretical yield toward 100% [20].

In practice, however, implementation of DKR for amino alcohols is often limited by the compatibility between the enzymatic and chemical catalytic components. Metal-catalyzed racemization may require elevated temperatures, strongly basic conditions, or organic reaction media that are not necessarily compatible with the conformational stability and catalytic activity of lipases or acylases. Conversely, conditions favorable for enzymatic activity may not provide sufficiently rapid racemization. This mismatch in optimal reaction conditions can substantially narrow the operating window of chemoenzymatic DKR systems and complicate their scale-up [20,23].

These limitations have motivated a broader shift away from the conventional “synthesize a racemate and then resolve it” paradigm toward direct asymmetric synthesis. Highly stereoselective biocatalysts such as AmDHs and transaminases (TAs) provide attractive alternatives because they establish the desired stereocenter directly from achiral or prochiral precursors. AmDHs catalyze asymmetric reductive amination of carbonyl compounds and can directly furnish highly enantioenriched chiral amines and amino alcohols [24]. TAs, in contrast, catalyze asymmetric transamination and provide another efficient route for converting prochiral ketones into optically enriched amines [25]. By avoiding the initial formation of a racemate, these strategies can circumvent the intrinsic 50% yield ceiling of kinetic resolution and directly afford highly enantioenriched products, including products with ee values exceeding 99% under suitable reaction conditions [24,25].

## 4. Direct Asymmetric Catalytic Synthesis of (*S*)-2-Aminobutanol

To overcome the intrinsic limitations of conventional chemical synthesis, particularly the 50% theoretical yield ceiling associated with racemate resolution and the generation of substantial amounts of saline waste, modern synthetic chemistry and biocatalysis have increasingly shifted toward direct stereoselective synthesis. Two major strategies are particularly attractive for industrial application: the stereoretentive reduction of readily available chiral amino acids to the corresponding amino alcohols [26], and the asymmetric amination of prochiral carbonyl compounds catalyzed by AmDHs or ω-TAs [24].

Among these strategies, the direct reduction of L-2-aminobutyric acid (L-ABA), which can be produced on a large scale by microbial fermentation, represents an attractive route to (*S*)-2-aminobutanol. Early chemical reduction systems mainly relied on excess reducing reagents such as NaBH_4_/H_2_SO_4_, resulting in the formation of stoichiometric by-products and consequently increasing the environmental factor (E-factor) of the overall process [27]. More recently, substantial progress has been made in the direct catalytic hydrogenation of amino acids using heterogeneous transition-metal catalysts. Tamura et al. developed a Rh-MoO_x_/SiO_2_ catalyst capable of directly hydrogenating various amino acids to the corresponding amino alcohols in water while largely preserving their optical purity [28]. For L-2-aminobutyric acid, the reaction achieved >99.9% conversion and a 90.6% yield for the corresponding amino alcohol. However, the stereochemical configuration and ee of the product derived specifically from L-2-aminobutyric acid were not determined in that study. The enhanced catalytic performance was attributed primarily to the synergistic interaction between Rh and MoO_x_, which markedly improved catalytic activity and selectivity [28].

**Figure 2:**
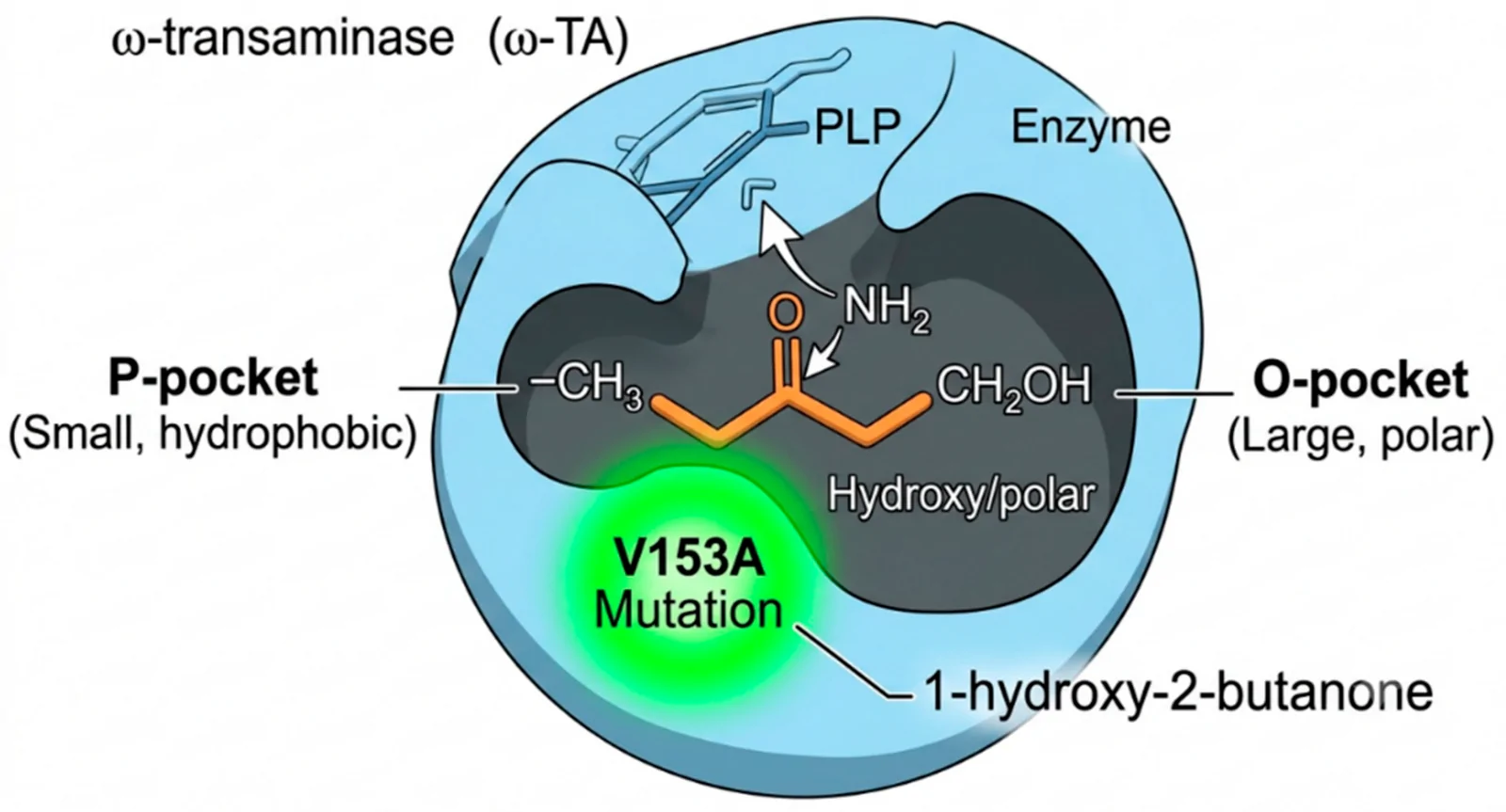
Stereoselectivity-induction model of the dual-pocket active site (P-pocket/O-pocket) of ω-transaminases, with 2-ketobutyrate (the precursor to L-2-ABA, the upstream chiral amino acid) shown as the substrate in the binding pocket. The V153A mutation is shown as an example of pocket-enlarging engineering.

Biocatalytic asymmetric amination provides another important platform for the stereoselective synthesis of chiral amines and their precursors. ω-TAs generally possess a characteristic dual-pocket architecture, comprising a small pocket that accommodates methyl-sized substituents and a larger pocket that accommodates bulkier groups (Figure 2), thereby contributing to their high substrate and stereochemical selectivity [29]. It should be noted, however, that the OATA and ppTA variants reported in studies [30,31] catalyze the formation of L-2-aminobutyric acid (L-ABA) from 2-ketobutyrate (Figure 2), not the direct asymmetric transamination of 1-hydroxy-2-butanone to (*S*)-2-aminobutanol. These enzymes are therefore more appropriately considered catalysts for the upstream biosynthesis of the chiral L-ABA precursor, which can subsequently be reduced to the corresponding amino alcohol (*S*)-2-aminobutanol. The direct ω-TA-catalyzed conversion of 1-hydroxy-2-butanone to (*S*)-2-aminobutanol—although chemically plausible given the dual-pocket model—has comparatively limited published support and represents an attractive target for future enzyme engineering.

Zhang et al. engineered an (*S*)-selective ω-TA from *Ochrobactrum anthropi* (OATA) and obtained the double mutant OATA^L57C/M419I^ with substantially enhanced catalytic efficiency toward α-ketobutyric acid. When incorporated into a threonine deaminase/OATA cascade with 300 mM L-threonine as the starting substrate, the engineered enzyme enabled 94% conversion to L-ABA within 12 h, with the product exhibiting >99% ee [30]. This approach provides an efficient route for generating enantiomerically enriched L-ABA as a precursor for subsequent synthesis of (*S*)-2-aminobutanol. Similarly, Zhang et al. reported the ppTA-V153A variant derived from *Paracoccus pantotrophus*. The V153A substitution reduced steric hindrance around the substrate-binding region and slightly enlarged the substrate-binding pocket, thereby facilitating substrate access and improving catalytic efficiency. Molecular docking and molecular dynamics analyses supported these structural effects. The engineered enzyme tolerated 2-ketobutyrate concentrations of up to 300 mM and maintained a conversion rate of 74% for the production of L-ABA [31]. Together, these studies demonstrate that protein engineering of ω-TAs can substantially enhance the high-substrate-loading synthesis of L-ABA, thereby strengthening the upstream biocatalytic supply of this chiral precursor. They do not, however, demonstrate the direct enzymatic conversion of 1-hydroxy-2-butanone to (*S*)-2-aminobutanol.

**Figure 3:**
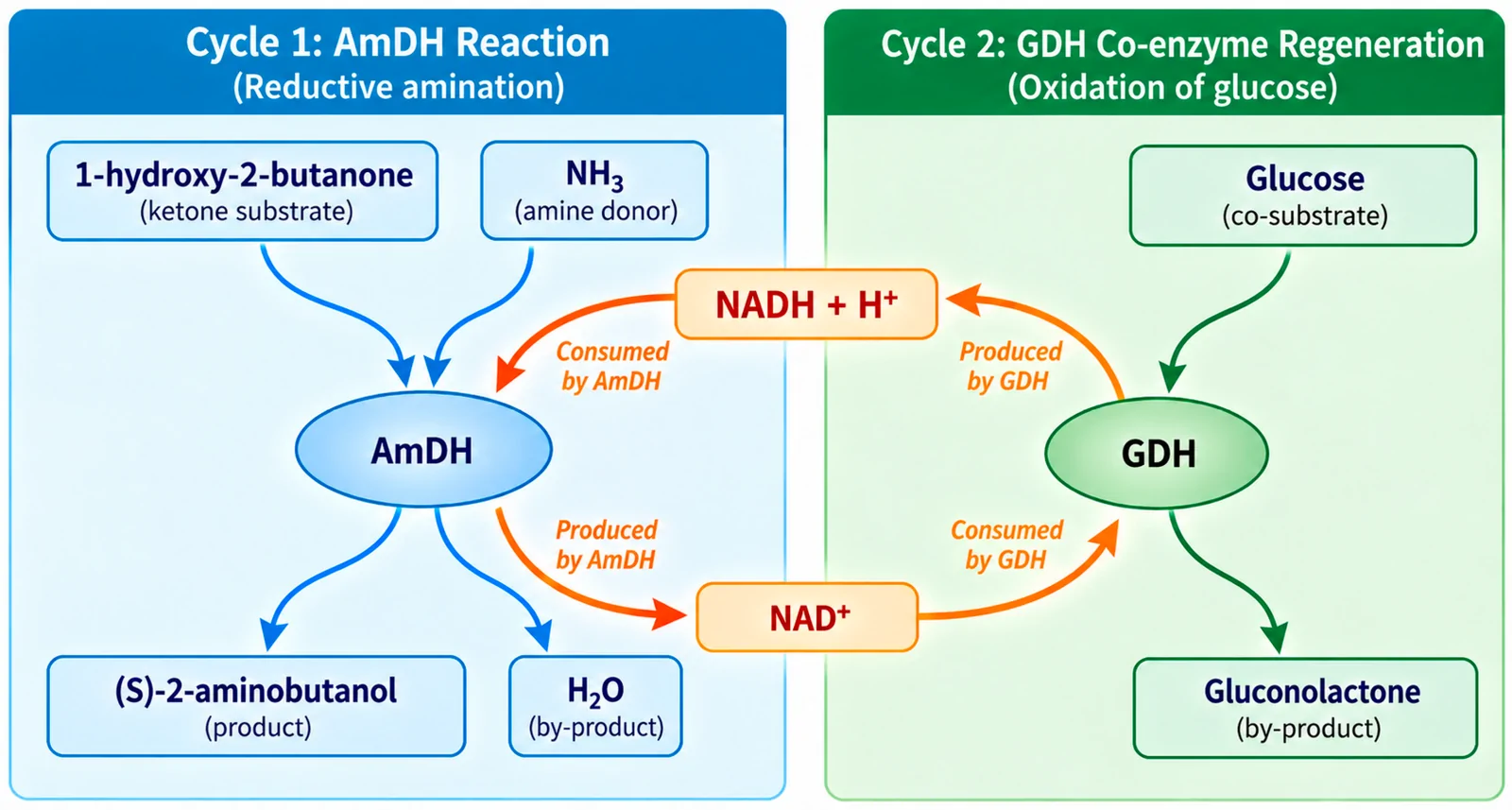
Schematic illustration of the AmDH-GDH coupled catalytic system for (*S*)-2-aminobutanol synthesis with NADH/NAD^+^ recycling. AmDH catalyzes the reductive amination of 1-hydroxy-2-butanone using ammonia and NADH to produce (*S*)-2-aminobutanol. Simultaneously, GDH catalyzes glucose oxidation to regenerate NADH from NAD^+^. The orange arrows indicate the NADH/NAD^+^ recycling loop linking the two enzymatic reactions.

Compared with transaminases, AmDHs offer the industrial advantage of directly using aqueous ammonia as the nitrogen source while avoiding the generation of stoichiometric amine-donor-derived coproducts [32]. Chen et al. reshaped the active-site pocket of leucine dehydrogenase-derived LfAmDH, generating engineered variants with an enlarged substrate-binding cavity that exhibited excellent enantioselectivity (>99% ee) toward chiral amine products [33]. Tong et al. further reported an engineered AmDH variant, wh84, which achieved conversion rates of 91–99% at substrate concentrations of 100–200 mM [34]. To address unfavorable thermodynamic equilibria and the high cost associated with cofactor regeneration in multienzyme biocatalysis, several closed-loop cascade systems have also been developed (Figure 3). In an “ω-TA + LDH + FDH” cascade, lactate dehydrogenase (LDH) removes the coproduct pyruvate, while formate dehydrogenase (FDH) regenerates NADH through the oxidation of formate, thereby shifting the reaction equilibrium toward product formation [35]. In an “AmDH + GDH” coupled system, Xie et al. reported the continuous production of (*S*)-2-aminobutanol using co-immobilized AmDH and glucose dehydrogenase (GDH) in a continuous-flow biocatalytic process. The system exhibited high operational stability during continuous processing, achieving a space-time yield (STY) of 124.5 g·L^−1^·d^−1^ and shortening the production cycle by more than 60% compared with the conventional batch process [36].

From an industrial perspective, direct asymmetric synthesis offers clear advantages over conventional kinetic-resolution processes. In terms of theoretical yield, asymmetric synthesis of a prochiral substrate can, in principle, achieve a maximum yield of 100%, in contrast to the intrinsic 50% yield ceiling of conventional kinetic resolution [37,38]. In addition, biocatalytic asymmetric synthesis is generally regarded as an environmentally favorable alternative because of its high stereoselectivity, mild reaction conditions, and reduced dependence on stoichiometric chiral resolving agents [38]. Nevertheless, quantitative claims regarding reductions in waste-salt generation require specific process-level evidence and should not be generalized without direct experimental or industrial data. From an economic perspective, advanced cofactor-regeneration strategies allow expensive cofactors such as NADH or NADPH to be supplied only in catalytic amounts, thereby substantially reducing cofactor-related material costs during subsequent process scale-up [39].

The suitability of in vitro multienzyme cascades and whole-cell systems for (*S*)-2-aminobutanol production depends on their respective advantages and limitations (Table S1). Whole-cell systems offer cost-effective enzyme production, endogenous cofactor regeneration, and scalable fermentation, as demonstrated by the production of 45.3 g·L^−1^ L-2-aminobutyric acid (L-2-ABA) in engineered *E. coli* [40]. However, intermediate toxicity, competing metabolic pathways, and potential product inhibition constrain their efficiency [41,42]. In contrast, in vitro cascades provide greater control over reaction conditions and stereoselectivity, with co-immobilized continuous-flow systems achieving space–time yields of up to 124.5 g·L^−1^·d^−1^ in related biocatalytic systems [36]. Nevertheless, enzyme production costs, cofactor requirements, and scale-up challenges remain significant barriers.

A hybrid strategy combining whole-cell fermentation for L-2-ABA precursor production with in vitro enzymatic conversion to (*S*)-2-aminobutanol may offer a practical route toward industrialization. Such an approach could integrate the scalability and cost efficiency of microbial fermentation with the catalytic flexibility of cell-free systems. However, efficient downstream conversion and cofactor management require further optimization, while direct de novo whole-cell production remains a promising long-term objective.

## 5. Synthetic Biology-Based Whole-Cell and Multienzyme Cascade Cell Factories

Over the past decade, advances in synthetic biology have expanded the production of chiral chemicals from conventional in vitro single-enzyme catalysis toward whole-cell and multienzyme cascade cell factories based on microbial chassis [34]. The use of chassis organisms such as *Escherichia coli* and *Saccharomyces cerevisiae* to synthesize target chiral amino alcohols in situ from inexpensive carbon sources or bulk amino acids represents a promising green manufacturing strategy for pharmaceutical intermediates [43].

**Figure 4:**
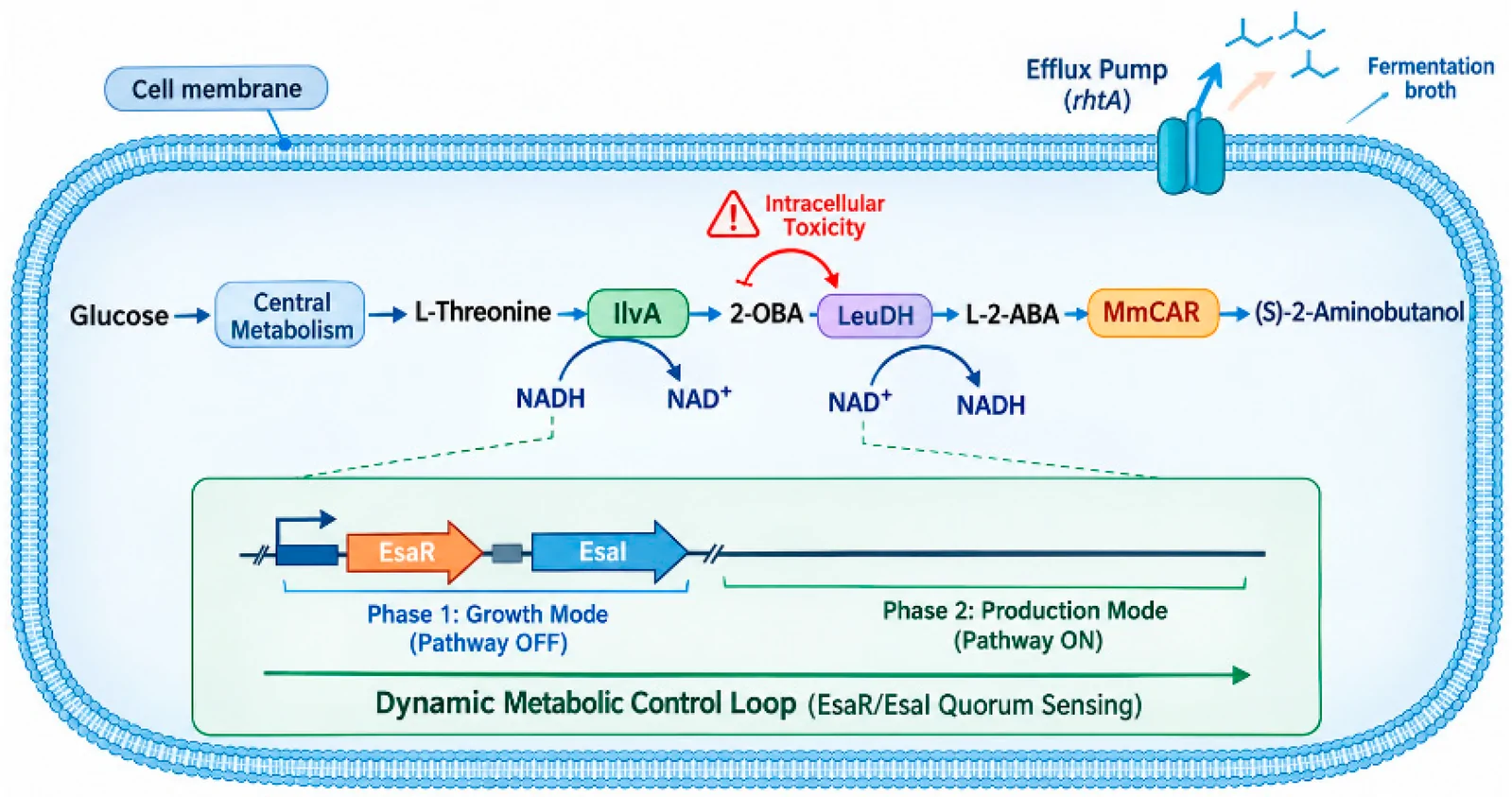
Schematic overview of heterologous metabolic network reconstruction and dynamic engineering regulation for de novo synthesis of (*S*)-2-Aminobutanol.

The construction of efficient whole-cell factories requires overcoming the constraints imposed by native metabolic networks and establishing efficient non-natural biosynthetic cascades [43]. For the de novo synthesis of (*S*)-2-aminobutanol and its precursors, a representative engineered pathway starts from intracellularly available L-threonine or, further upstream, glucose. In the eukaryotic chassis *S. cerevisiae*, researchers constructed a complete multienzyme cascade from L-threonine to (*S*)-2-aminobutanol (Figure 4) [44,45]. In this pathway, threonine deaminase (SlTD or BsIlvA) first catalyzes the deamination of L-threonine to generate the key intermediate 2-oxobutyrate (2-OBA). Subsequently, a mutant *E. coli* glutamate dehydrogenase (EcGDH′) catalyzes the asymmetric reductive amination of 2-OBA to form (*S*)-2-aminobutyric acid (L-2-ABA) [45]. To further reduce the carboxyl group, a carboxylic acid reductase from *Mycobacterium marinum* (MmCAR) was introduced, followed by an *E. coli* aldehyde reductase in the downstream step, ultimately enabling the de novo intracellular synthesis of (*S*)-2-aminobutanol [45]. In the prokaryotic *E. coli* chassis, recent de novo fermentation strategies have employed high-L-threonine-producing strains and optimized threonine deamination and reductive amination to efficiently synthesize L-2-ABA [40]. Earlier work similarly demonstrated total biosynthesis of L-2-ABA, also referred to as L-homoalanine, from glucose by engineering threonine supply and evolving glutamate dehydrogenase activity toward 2-OBA; notably, L-2-ABA can subsequently serve as a chiral precursor for (*S*)-2-aminobutanol [6].

Despite the potential advantages of whole-cell factories, their transition toward industrial high-cell-density fermentation remains constrained by several metabolic engineering challenges. One major limitation is the toxicity of the key intermediate 2-OBA. Because 2-OBA is structurally similar to pyruvate, its excessive accumulation can interfere with pyruvate metabolism and consequently impair cell growth [40,41]. In engineered *E. coli*, increasing 2-OBA concentrations caused a dose-dependent reduction in biomass, while 10 g·L^−1^ 2-OBA reduced biomass to approximately 22% of the control level and decreased pyruvate dehydrogenase complex (PDC) activity by approximately 77% [41]. These observations led to the proposal that 2-OBA may competitively inhibit PDC and thereby perturb pyruvate metabolism. Product toxicity can impose an additional constraint on microbial production of amino alcohols. For example, intracellular accumulation of serinol, a highly polar amino alcohol, has been reported to inhibit host-cell growth at concentrations above approximately 100 mM, thereby limiting further increases in fermentative titer [42]. However, the extent and molecular basis of such toxicity are compound-specific and remain to be established specifically for (*S*)-2-aminobutanol.

A second major limitation is the intracellular availability and balance of reducing cofactors. Reductive reactions involved in amino acid and amino alcohol biosynthesis frequently depend on NADH or NADPH, and a mismatch between enzyme cofactor preference and intracellular cofactor availability can restrict pathway flux. Cofactor engineering can therefore substantially improve synthetic pathway performance. For example, engineering an NADPH-dependent 2-oxo-4-hydroxybutyrate reductase together with increased intracellular NADPH supply enhanced the yield of 2,4-dihydroxybutyrate by approximately 50% in engineered *E. coli*, demonstrating the importance of matching enzyme cofactor specificity with the intracellular redox environment [46]. Similarly, in L-2-ABA biosynthesis, LeuDH K72A requires NADH for the reductive amination of 2-OBA, and modulation of NADH regeneration was shown to affect product formation [40]. These findings indicate that cofactor engineering should focus on maintaining pathway-wide redox balance rather than simply maximizing a single reduced cofactor pool.

Recent studies have therefore adopted multilevel synthetic biology strategies to alleviate precursor toxicity and rebalance metabolic flux [47]. Transporter engineering represents one such strategy, but its effects strongly depend on pathway architecture. RhtA is an important L-threonine exporter in E. coli, and modulation of threonine efflux can substantially influence the intracellular precursor supply available for L-2-ABA biosynthesis [40]. In a recently engineered L-2-ABA-producing strain, deletion of rhtA increased L-2-ABA accumulation to 9.68 g·L^−1^, whereas simultaneous disruption of *rhtA* and *rhtC* reduced production, highlighting the importance of balancing precursor retention with cellular metabolic homeostasis [40]. More recent studies have similarly demonstrated that appropriate modulation of threonine transport can maintain a favorable intracellular precursor pool, whereas excessive precursor retention can cause metabolic stress [40]. These findings indicate that transporter engineering should be used to optimize intracellular metabolite homeostasis rather than simply maximizing metabolite export [48].

Engineering of pathway enzymes provides another strategy for reducing the accumulation of inhibitory intermediates. For example, remodeling the substrate-binding pockets of ω-transaminases or dehydrogenases can enhance catalytic conversion of 2-OBA. As discussed above, the V153A substitution in ppTA reduced steric hindrance and slightly enlarged the substrate-binding pocket, thereby enhancing catalytic performance toward 2-ketobutyrate [49]. Increasing downstream catalytic capacity can therefore potentially reduce the accumulation of inhibitory intermediates, although this effect should be experimentally validated in whole-cell systems rather than inferred solely from in vitro enzyme properties.

A particularly important advance has been the development of dynamic metabolic control. In the L-2-ABA-producing *E. coli* system, an Esa quorum-sensing (Esa-QS) circuit was introduced to dynamically regulate *ilvA* expression and temporally separate biomass accumulation from 2-OBA formation. At low cell density, EsaR represses expression of the target pathway, whereas accumulation of the EsaI-generated signaling molecule N-acyl-homoserine lactone (AHL) with increasing biomass relieves EsaR-mediated repression and activates 2-OBA synthesis. This growth-dependent regulatory architecture reduces premature accumulation of the toxic intermediate while preserving biomass formation. Combined with optimization of the 2-OBA-to-L-2-ABA conversion pathway, genome-scale model-guided carbon-flux redistribution, NADH regeneration, balanced L-threonine transport, dynamic control of 2-OBA consumption, and transcriptional regulator engineering, the final *E. coli* strain ABA40 produced 45.3 g·L^−1^ L-2-ABA with a yield of 0.31 g/g glucose after 72 h of fed-batch fermentation [40]. It should be noted explicitly that this titer refers to the upstream chiral amino acid precursor L-2-ABA, not to the final amino alcohol product (*S*)-2-aminobutanol. Direct whole-cell titers of (*S*)-2-aminobutanol itself remain less extensively reported; current best performance for (*S*)-2-aminobutanol production is dominated by in vitro multienzyme cascades (Section 4), with STYs exceeding 100 g·L^−1^·d^−1^ in continuous-flow co-immobilized systems [36]. The downstream enzymatic reduction of L-2-ABA to (*S*)-2-aminobutanol (e.g., via Rh-MoO_x_/SiO_2_ hydrogenation [28] or carboxylic acid reductase cascades [45]) remains an important and only partially optimized bridge between the two product stages.

Dynamic metabolic regulation has also been successfully implemented in other microbial chassis. For example, a growth phase-dependent autonomous bifunctional genetic switch was used in *Corynebacterium glutamicum* to switch metabolic flux from growth mode to production mode, resulting in high-level GABA production and illustrating the broader applicability of autonomous dynamic control in microbial cell factories [49].

## 6. Conclusions and Future Perspectives

The evolution of (*S*)-2-aminobutanol synthesis from conventional chemical routes toward modern biomanufacturing reflects a broader transition from energy-intensive and pollution-prone processes toward manufacturing strategies characterized by higher atom economy, greater selectivity, and improved environmental sustainability [11,15]. Conventional chemical routes, including the 1-nitropropane process involving high-temperature and high-pressure hydrogenation and the 1-butene epoxidation route limited by insufficient regioselectivity, face inherent challenges associated with process safety and the demanding separation of positional isomers [13,50]. In contrast, biocatalysis exploits the highly organized substrate-binding pockets of enzymes to achieve excellent stereoselectivity and, in asymmetric synthesis, can circumvent the intrinsic 50% theoretical yield ceiling associated with conventional kinetic resolution [5,9].

Nevertheless, current biomanufacturing technologies are still undergoing the transition from laboratory-scale proof of concept to economically competitive industrial implementation. The major challenge has shifted from establishing a functional biosynthetic pathway to optimizing metabolic flux and engineering cellular robustness. Key issues include overcoming substrate and product inhibition under high substrate loadings [31,32], reducing the cost and metabolic burden associated with cofactor regeneration [35,37], and alleviating the toxicity of pathway intermediates such as 2-OBA [40,41]. In particular, the pronounced growth-inhibitory effects associated with excessive 2-OBA accumulation demonstrate that precursor supply cannot be optimized independently of downstream conversion capacity. Instead, pathway flux must be coordinated across precursor generation, reductive amination, cofactor regeneration, and cellular growth.

Over the next five to ten years, deeper integration of synthetic biology with artificial intelligence (AI) is expected to accelerate progress in this field. Future cell factories are likely to evolve beyond simple constitutive overexpression of individual heterologous enzymes toward dynamically regulated systems capable of responding to changes in cell density, metabolic state, and intracellular metabolite concentrations. QS-based regulatory circuits and highly responsive metabolite biosensors could enable microbial chassis to temporally coordinate biomass accumulation with the formation of toxic intermediates, thereby reducing metabolic burden and improving pathway robustness [49]. The successful application of dynamic regulation to temporally separate biomass accumulation from precursor or product formation provides an important foundation for the development of more autonomous microbial production systems [40,49].

At the catalyst level, protein structure prediction tools such as AlphaFold, together with machine learning-assisted enzyme engineering and adaptive evolution strategies, are expected to accelerate the rational design and development of key pathway enzymes [51]. High-throughput protein engineering strategies such as combinatorial active-site saturation testing (CAST) may facilitate the development of highly evolved amine dehydrogenases and transaminases capable of functioning efficiently at substrate concentrations of several hundred millimolar while retaining high stereoselectivity [52,53]. Importantly, future enzyme engineering should focus not only on catalytic activity but also on substrate inhibition, thermostability, cofactor compatibility, and intracellular performance under the complex physicochemical conditions encountered during high-cell-density fermentation.

At the process scale, integration of reaction engineering with in situ separation will be important for further improving productivity. Conventional batch fermentation and biotransformation processes are increasingly being complemented by continuous-flow biocatalytic systems incorporating in situ product removal (ISPR) [36,54]. Coupling co-immobilized multienzyme cascades with microfluidic devices or packed-bed reactors (PBRs) may alleviate product inhibition, improve catalyst utilization, and increase space-time yield [55]. Cell-free multienzyme cascades may provide an additional alternative when intracellular product toxicity becomes a major limitation. For example, a recently developed methanol-to-serinol cascade circumvented intracellular serinol toxicity and achieved 43.86 mM (4 g·L^−1^) serinol from 150 mM methanol with an 87.7% carbon yield [42], illustrating the potential of combining alternative carbon feedstocks with cell-free biocatalysis.

In parallel, the broader development of green fine-chemical manufacturing is moving toward non-food feedstocks and lower-carbon production. Biosynthetic cascades based on methanol, CO_2_, or non-food lignocellulosic feedstocks may improve carbon utilization efficiency and reduce dependence on conventional sugar- and fossil-derived resources [54,56,57]. Further optimization of closed-loop cofactor recycling and efficient in situ cofactor regeneration should reduce auxiliary reagent consumption and waste generation, thereby improving the overall environmental performance of these processes [16,58,59,60].

Overall, the development of (*S*)-2-aminobutanol synthesis illustrates the progressive transition from conventional chemical processing toward increasingly selective and sustainable biomanufacturing. Although a substantial gap remains between laboratory-scale systems capable of achieving >99% ee and robust, economically viable production at industrial scale, continued advances in metabolic engineering of both eukaryotic and prokaryotic chassis are expected to narrow this gap [61]. The integration of enzyme engineering, dynamic metabolic regulation, redox balancing, synthetic biology, and process intensification may ultimately enable efficient production of (*S*)-2-aminobutanol and related chiral amino alcohols under mild conditions with substantially reduced dependence on fossil-derived feedstocks.

### 6.1. Ranking of Critical Industrialization Bottlenecks

The industrialization of (*S*)-2-aminobutanol production faces several critical challenges. The primary bottleneck is the toxicity of 2-oxobutyrate (2-OBA), which disrupts central carbon metabolism. At 10 g·L^−1^, exogenous 2-OBA reduces *E. coli* biomass to approximately 22% of the control and inhibits pyruvate dehydrogenase activity by approximately 77% [41]. Addressing this limitation requires dynamic pathway regulation or cell-free catalysis [40]. A second major challenge is the inefficient conversion of L-2-aminobutyric acid (L-2-ABA) to (*S*)-2-aminobutanol. Despite L-2-ABA titers exceeding 40 g·L^−1^, downstream conversion remains insufficiently optimized, with existing chemical and enzymatic routes requiring metal catalysts or ATP-dependent reactions [28,45]. Additional constraints include costly NAD(P)H regeneration, complex product separation, and insufficiently characterized product toxicity [42]. Collectively, these challenges highlight the need for integrated advances in metabolic regulation, enzyme engineering, and downstream processing.

### 6.2. AI-Assisted Approaches Targeting Specific Bottlenecks

Artificial intelligence (AI) offers promising tools to address these bottlenecks, although most applications remain to be validated specifically for (*S*)-2-aminobutanol production. First, machine learning and model predictive control could optimize dynamic regulatory circuits, such as quorum-sensing systems, to minimize 2-OBA accumulation and associated toxicity [40,49]. Second, AI-assisted protein structure prediction and enzyme engineering could facilitate the discovery and optimization of carboxylic acid reductases, aldehyde reductases, and related enzymes for efficient conversion of L-2-ABA to (*S*)-2-aminobutanol [45]. Third, machine-learning-guided cofactor specificity engineering could improve NAD(P)H utilization and intracellular redox balance. Finally, Bayesian optimization and reinforcement learning could support continuous-flow biocatalysis, enzyme immobilization, and in situ product removal to enhance productivity and operational stability [36]. While AI cannot directly eliminate intrinsic intermediate toxicity, its integration with metabolic engineering and process optimization may accelerate the development of economically viable (*S*)-2-aminobutanol biomanufacturing.

## Figures and Tables

**Table 1:** Comparison of crystallization-based chemical resolution and biocatalytic kinetic resolution of racemic 2-amino-1-butanol.

Resolution Strategy	Representative Resolving Agent/Catalyst	Typical Process Features	Major Limitations	Primary Reference(s)
Crystallization-based chemical resolution	L-Tartaric acid	Diastereomeric salt formation followed by selective crystallization, salt liberation, extraction, and solvent recovery	Theoretical yield of the desired enantiomer ≤ 50% without racemization; relatively high consumption of acids, bases, solvents, and energy; generation of salt-containing waste streams	[16,17]
Lipase-catalyzed kinetic resolution	Enantioselective lipase	Selective N- or O-acylation in organic media using an appropriate acyl donor	Intrinsic 50% theoretical yield limit for conventional KR; organic-solvent use and downstream separation remain necessary	[18,19,20]
Acylase-catalyzed kinetic resolution	Immobilized penicillin G acylase (PGA)	Resolution of derivatized substrates such as N-phenylacetyl derivatives; applicable to immobilized-enzyme and continuous-flow operation	Conventional KR remains subject to the 50% theoretical limit; substrate derivatization introduces additional process steps; integration with racemization may present catalyst-compatibility challenges	[21,22,23]

## Data Availability

No new data were created or analyzed in this study. Data sharing is not applicable to this article.

## Supplementary Materials

The following supporting information can be downloaded at: https://www.mdpi.com/article/10.3390/ijms27209203/s1.
