# Supplementary material for: Evolution of (S)-2-Aminobutanol Synthesis from Chemical Resolution to Biomanufacturing
Source: Int J Mol Sci. 2026 Oct 16;27(20):9203. doi: 10.3390/ijms27209203 (PMC13705519; doi:10.3390/ijms27209203)
Supplement: Supplementary file 1 [file ijms-27-09203-s001.zip › ijms-4612044-supplementary.pdf]

Table S1. Quantitative comparison of major *(S)*-2-aminobutanol production strategies

Strategy	Platform	Substrate concentration	Conversion / yield	ee (%)	STY ($g \cdot L^{-1} \cdot d^{-1}$)	Major process limitation	Primary reference(s)
1-Nitropropane route (Henry + hydrogenation)	Chemical	NR	NR (racemic intermediate)	n/a (requires downstream resolution)	NR	High-pressure H_2; safety; no intrinsic stereocontrol	[10,11]
1-Butene → 1,2-epoxybutane route	Chemical	NR	Variable; regioisomer mix 60:40–85:15	n/a (requires downstream resolution)	NR	Regioselectivity at ring opening; downstream isomer separation	[12–14]
L-Tartaric-acid crystallization resolution	Chemical	NR	≤50% theoretical (of desired enantiomer)	>99 (after multi-cycle recryst.)	NR	50% yield ceiling; high salt waste; multiple recryst.	[17,18]
Lipase-catalyzed KR	*In vitro* / enzyme	Variable	≤50% (conventional KR)	>99 (high selectivity)	Variable	50% ceiling; organic solvent; downstream separation	[19,22,23]
Acylase (PGA)-catalyzed KR	*In vitro* / immobilized enzyme	Variable	≤50%	>99	High in continuous-flow packed-bed	50% ceiling; substrate derivatization	[20,21,24]
ω-TA cascade (threonine → L-2-ABA → reduction)	*In vitro* multienzyme	300 mM (L-threonine)	94% (L-ABA step)	>99	Variable	Cofactor regeneration; L-ABA → (*S*)-2-aminobutanol bridge	[31]
AmDH + GDH co-immobilized continuous flow	*In vitro* multienzyme (continuous)	100–200 mM (2-OBA)	91–99%	>99	124.5	Cofactor cost; enzyme stability over long operation	[35,37]
E. coli whole-cell *de novo* (L-2-ABA)	Whole-cell (fed-batch)	Glucose fed-batch	0.31 g/g glucose (yield); 45.3 $g \cdot L^{-1}$ (L-2-ABA)	>99	~15 (L-2-ABA)	2-OBA toxicity; intracellular redox balance	[44]
E. coli whole-cell *de novo* [*(S)*-2-aminobutanol]	Whole-cell (fed-batch)	Glucose fed-batch	NR for direct final product	>99 (expected)	NR	Downstream *(S)*-2-aminobutanol-specific toxicity; 2-OBA stress	[42,43]

Notes: "NR" indicates that the specific quantitative metric has not been reported for the specific target molecule in the cited literature; data should be interpreted as the closest available proxy. ee = enantiomeric excess; STY = space-time yield.
